# COPASAAR – A database for proteomic analysis of single amino acid repeats

**DOI:** 10.1186/1471-2105-6-196

**Published:** 2005-08-03

**Authors:** Daniel P Depledge, Andrew R Dalby

**Affiliations:** 1 Schools of Biological and Chemical Sciences and Engineering, Computer Science and Mathematics, Washington Singer Laboratories, University of Exeter, Prince of Wales Road, Exeter, EX4 4PS UK

## Abstract

**Background:**

Single amino acid repeats make up a significant proportion in all of the proteomes that have currently been determined. They have been shown to be functionally and medically significant, and are associated with cancers and neuro-degenerative diseases such as Huntington's Chorea, where a poly-glutamine repeat is responsible for causing the disease. The COPASAAR database is a new tool to facilitate the rapid analysis of single amino acid repeats at a proteome level. The database aims to simplify the comparison of repeat distributions between proteomes in order to provide a better understanding of their function and evolution.

**Results:**

A comparative analysis of all proteomes in the database (currently 244) shows that single amino acid repeats account for about 12–14% of the proteome of any given species. They are more common in eukaryotes (14%) than in either archaea or bacteria (both 13%). Individual analyses of proteomes show that long single amino acid repeats (6+ residues) are much more common in the Eukaryotes and that longer repeats are usually made up of hydrophilic amino acids such as glutamine, glutamic acid, asparagine, aspartic acid and serine.

**Conclusion:**

COPASAAR is a useful tool for comparative proteomics that provides rapid access to amino acid repeat data that can be readily data-mined. The COPASAAR database can be queried at the kingdom, proteome or individual protein level. As the amount of available proteome data increases this will be increasingly important in order to automate proteome comparison. The insights gained from these studies will give a better insight into the evolution of protein sequence and function.

## Background

Single amino acid repeats (SAARs) are uninterrupted runs of identical amino acids that exist in many proteins and are currently a major focus of research. These are an example of a simple sequence repeat (SSR), which occurs when a simple sequence motif is repeated in the DNA sequence. These repeats are found in the proteome and can eventually dictate the structure and function of proteins. Repeats within the amino acid sequence are usually dependent on repetitive elements in the genome. They originate from unequal crossing-over or replication errors resulting from the formation of unusual DNA secondary structures such as hairpins or slipped strands [1-3]. Amongst the various DNA duplication events, SSRs are abundant in eukaryotic genomes and may be a major source of quantitative genetic variation [4-6]. SSRs in the codingregions of proteins can give rise to a variety of repeats including SAARs, short tandem repeats, and the repetition of homologous domains of 100 or more residues. However the focus of this work is solely on SAARs.

There has been some suggestion that these repeated sequence patterns may be a mechanism that provides regular arrays of spatial and functional groups, useful for structural packing or for one to one interactions with target molecules [7]. This suggests that error-prone SAAR expansion allows the rapid evolution of proteins with repetitive structure, which can lead to rapidly changing phenotypes [8].

Marcotte *et al*., suggested that eukaryotic proteomes have a significantly higher incidence of SAARs than either bacterial or archaeal proteomes [9]. They showed that most SAARs occur in protein classes associated only with eukaryotes so protein classes associated with both eukaryotes and prokaryotes are much less likely to contain repeats. This would imply that the formation of SAARs is a relatively recent evolutionary event.

What is interesting is that SAARs can be either functionally significant or extremely pathogenic depending on the proteins involved. Several human inherited neurodegenerative diseases are triplet-repeat diseases associated with proteins containing long runs of glutamine (long CAG codon iterations which result from mutations of SAARs) as shown in Table 1[10]. The severity of these diseases seems can be correlated with the extent of iterations of the CAG codon above a certain threshold [11]. Also notable is that most of these proteins contain two or more additional long runs of amino acids other than glutamine [12]. Pathogenicity is due to inflammatory brain responses, oxidative damage and protein aggregations that clog the proteosome [13].

**Table 1:** Dominantly inherited neurodegenerative diseases are associated with abnormally expanded tracts of glutamine residues.

**Disease protein**	**Gln repeats**	**Other notable repeats/comments**
Huntington's disease protein	1 SAAR (23-residues)	2x Pro repeats (11-and 10-residues) 2x Glu repeats (6-and 5-residues)
Spinocerebellar ataxin type 1	2 SAARs (15-and 12-residues)	The two Gln repeats are separated by 4 residues
Androgen receptor (Kennedy's disease)	3 SAARs (21-, 6-and 5-residues)	1x Pro repeat (8-residues) 1x Ala repeat (5-residues) 1x Gly repeat (24-residues)

Legend: These SAARs are often accompanied by at least 2 other long SAARs of different amino acids. Gln – Glutamine, Pro – Proline, Ala – Alanine, Gly – Glycine, Glu – Glutamic acid.

Examples of functional SAARs can be seen in proteins which are associated with development and transcriptional regulatory capacities, with the majority of them active in central or peripheral nervous system function and development [14]. This has been extensively studied in *Drosophila melanogaster *but there are also examples in other eukaryotes, for example, the case of the transcription factor II (TFII) in humans [15] which contains a 34 residue glutamine run. This SAAR is absent from all related proteins, and yet appears to be functionally important. Extended runs can also provide substrates for caspase cleavage, yielding tangles, plaques, dead neurons and triggering apoptosis [16]. They also provide binding sites for protein-protein interactions [14].

SAARs are generally less than 20 residues long and are primarily composed of the residues of the amino acids glutamine, asparagine, serine, threonine, proline, histidine, glycine, alanine, aspartic acid and glutamic acid [17,18]. It is curious that glutamine followed by asparagine and serine are the most common SAARs found, especially when considering that the occurrence of leucine, isoleucine, alanine and valine in proteins is much greater. This is particularly interesting when considering that long SAARs of these 4 amino acids are rarely found.

The greatest challenge facing scientists who wish to study SAARs is the lack of tools for analysing SAARs and mining the data collected. While some software exists [19] for detecting and analysing SAARs, it is limited in its application in that it is only designed for analysing single proteins rather than whole proteomes. The aim of this paper is to describe a new web application dedicated to the analysis of SAARs in whole proteomes.

## Construction and content

The COPASAAR (COmparative Proteome Analysis of Single Amino Acid Repeats) database was developed in MySQL 4.0.18 running on Mandrake Linux version 10.0. Access to the database is through a web interface written in Perl:CGI and uses the Perl ChartDirector [20] and Descriptive::Statistics modules to generate histograms and statistical analysis of the data. Currently the database contains 244 proteomes, which are made up of 862,886 proteins with a storage requirement of 1.2 Gbytes.

### Repeat analysis software

Proteome data files were obtained from the integr8 database at the EBI [21] in Fasta format. These files were analysed for repeats using a series of scripts written in Perl. The database itself was written in SQL and the data was imported into the database as tab delimited text files using the mysqlimport client. This process was automated by the use of shell scripts.

The algorithm used for detecting and measuring a repeat compares each residue with the next one. If it finds two identical residues side-by-side then it continues the comparison to the next residue until it encounters a different amino acid. If a different residue is detected the programme records the repeat in an array of amino acid type and repeat length.

### Expected repeat lengths

As a reference to the actual occurrence of SAARs a statistical model was created where the amino acids are assumed to be distributed randomly based on their occurrence in a specific protein [22]. The probability of a SAAR of length n occurring will then be;

*P*(SAAR of length n) = *f*^*n *^(1 - *f*)^2^

Where f is the frequency of the particular amino acid in the protein. The (1-f)^2^. term accounts for there being a different amino acid at each end of the SAAR.

To find the expected number of repeats of a given amino acid within a protein this probability is multiplied by the number of potential starting points for the repeat. This will be equal to the sequence length minus the length of the repeat plus one.

Expected number of repeats of length n = *f*^*n *^(1 - *f*)^2 ^(*l *- (*n *- 1))

Where *l *is the length of the protein.

### Running times for the software

For all of the currently available proteomes the running time to extract all of the repeats and to generate the expected repeat tables at the protein and proteome level is about 3 hours on a Pentium 4 2.0 GHz. Import of the tables into MySQL is very rapid and takes less than 30 minutes. All of the scripts used to create the database can be downloaded from the COPASAAR website.

### The database schema

**Figure 1:**
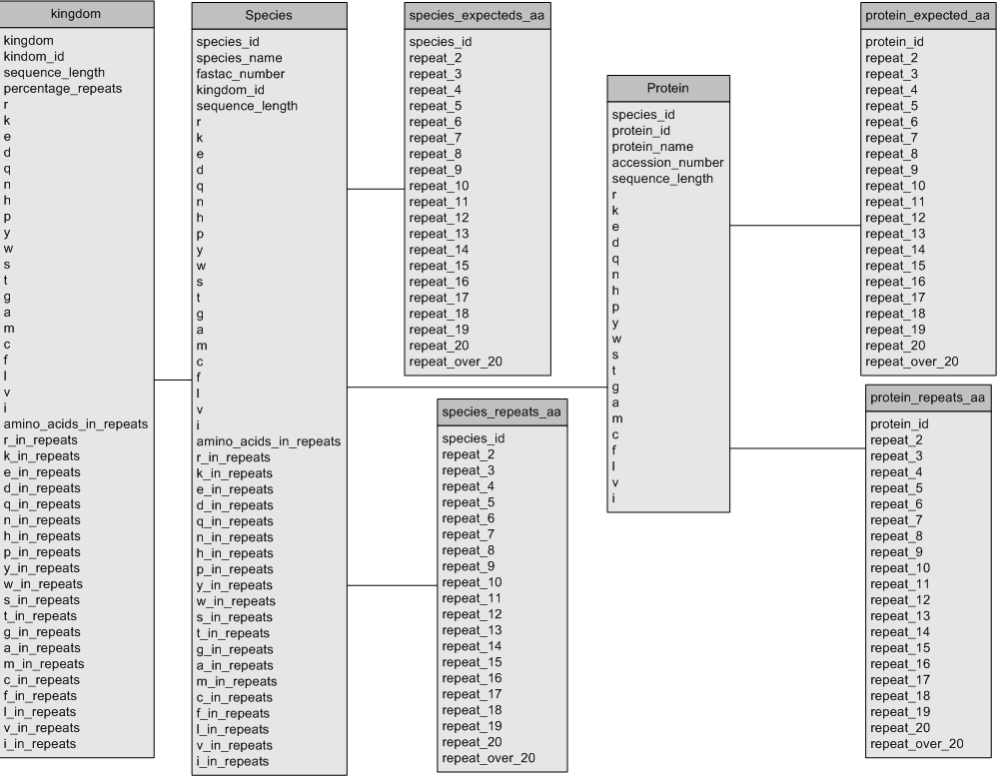
Database schema for COPASAAR. Note that each of the species_repeats, species_expected, protein_repeats and protein_expected tables will be repeated 20 times once for each amino acid.

The database contains 83 tables, most of which contain amino acid specific data. The database schema and table structure are shown in Figure 1. The data is stored at three different levels. At the individual protein, proteome and kingdom levels. While this means there is some redundancy of data this is required to speed up searches so that the amount of analysis that needs to be performed by a query is reduced. For example the expected frequencies of repeats could be calculated during a query from the amino acid occurrences in the proteins, but if this query is at the proteome level this would have to be done for all of the proteins within that proteome and then these would have to be summed to give a proteome level expectation. This would be less efficient and would result in slow querying of the database.

### COPASAAR website

The COPASAAR website houses the user interface to the main programmes, a documentation page featuring software documentation, and a download section so that users can download the database for use on local machines. The user-interface provides menu driven query access to the database. The user simply selects the species they wish to analyse and uses the 'post' method to send the request. Results are displayed either in tabulated or graphical form as bar charts. The website is hosted on an Apache (version 2.0.44) webserver.

### COPASAAR proteome data

The current database consists of 244 proteomes; 19 eukaryotic species, 205 prokaryotic species and 20 archaeal species. A full list of the species can be found in additional file 1.

## Utility

There have been previous systematic studies of simple amino acid repeat distributions in proteomes [7,14,23,24] but what COPASAAR aims to do is to provide a comprehensive and simple to update resource that means that makes comparative studies much easier to carry out and which also increases the number of biological questions that can be asked.

Adding new proteomes to the database is simple using the repeat analysis scripts and this procedure will be made even easier by the new naming convention for proteome data files that will use the organism name rather than using taxonomic identifiers that can change for the same proteome between database releases.

**Figure 2:**
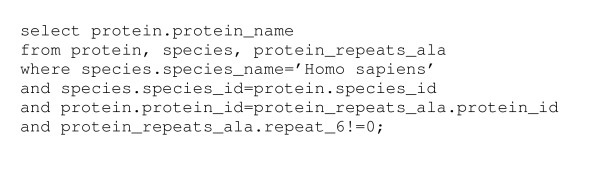
Example SQL script used to query the database for all proteins in humans with an alanine repeat of 6 amino acids.

Access to the database can be either through the web interface or for more experienced users the database can be queried directly using SQL. Accessing the MySQL database directly using SQL allows almost any query to be performed. Figure 2 shows the query to find all proteins with a repeat of 6 alanine residues from the human proteome. The problem with accessing the database this way is that it requires a working knowledge of SQL and also the structure of the database. For this reason the web interface will remain the preferred mode of interaction for novice or infrequent users. The web interface currently contains a set of simple queries that can be rapidly expanded depending on requirements. It is expected that users who download the database will want to implement queries specific to their own research which can be done by customising existing template scripts that generate SQL queries and that format the output either as tables or graphically to be displayed as webpages.

To illustrate some of the capabilities of the database using the current web interface functionality we have made a high level comparison of occurrence of SAARs across the three super kingdoms.

## Discussion

### Comparison of archaeal, eukaryotic and bacterial kingdoms

The mean number of amino acids within SAARs (as a percentage of the proteome) within the three super kingdoms is greatest in the eukaryotes at 14.34%. The archaea mean is 13.34% while bacterial proteomes are the lowest with a mean of 13.05% (Table 2a). The overall mean is 13.18%. Of the 19 eukaryotic species, 18 of these proteomes (95%) contain a greater percentage of SAARs than the overall mean compared to 8 out of 20 of the archaea (42%) and 65 out of 205 of the bacteria (32%) (Table 2). If however you look at the maximum length of the repeats between the three kingdoms the distinction between the eukaryotes and the archaea and bacteria is much clearer. In eukaryotes repeats over 20 amino acids occur in most species so far sequenced, although they are less common in the yeasts, whereas for bacteria repeats over 20 residues in length only occur exceptionally in certain *Vibrio *species (the seafood associated pathogens) and in *Lactobacillus plantarum*. In archaea a glycine repeat over 20 residues only occurs in *Haloarcula marismortui*. These results supports the finding of Marcotte [9] and also suggest that the differences between the kingdoms can be specified in terms of repeats for a few amino acids. Glutamine repeats are particularly characteristic of eukaryotes where they have a long tailed distribution, which in the mammals and plants extends beyond 20 residues. It is of particular clinical interest that glutamine repeats play a significant role in eukayotic proteomes because they are associated with amyloid plaque formation in diseases such as Huntington's chorea and spinocerebellar ataxia [25-27]. A functional explanation for the occurrence of glutamine repeats in transcription factor genes has been suggested by Fondon *et al*. and this could be the main contributing factor to the occurrence of these repeats in eukaryotes [8]. The only eukaryotes where glutamine does not form the longest repeat are *Plasmodium falciparum, Arabidopsis thaliana *and *Caenorhabditis elegans*. *A. thaliana *contains a very characteristic long lysine repeat (over 100 amino acids), while in *C. elegans *the longest repeat is serine.

**Table 2:** The proportion of a proteome composed of SAARs and the percentage of proteomes in each kingdom with a greater number of SAARs than the mean. *The overall mean is 13.18%

**Kingdom**	**SAARs **(as a percentage of the whole proteome)	**Proteomes **(with a greater % of repeats than the overall mean*)
Eukaryotes	14.5%	95%
Archaea	13.3%	45%
Bacteria	13.1%	32%

*P. falciparum*, has a very unusual repeat distribution that is different to all other proteomes, prokaryotes and archaea included. Nearly 20% of the *P. falciparum *proteome is made up of repeats. The distribution of asparagine repeats is particularly significant. There are 137 repeats of over 20 asparagines in length which is highly unusual as long asparagine repeats are associated with prion domains and fibril formation [28,29]

The amino acid compositions of SAARs across the kingdoms are shown in Table 3. The eukaryotes, feature leucine, serine and glutamic acid as the top three constituents. Archaea features leucine and glutamic acid as its top two constituents, while bacteria feature leucine and alanine as the top two constituents. These results agree with the overall distributions of amino acids in the three kingdoms, but although leucine appears in many short repeats and so makes a large contribution to the number of amino acids in SAARs it very rarely has long repeats in any proteome and it is the longest repeat in only a few bacterial species.

**Table 3:** SAARs composition by amino acid.

**Amino Acid**	**Eukaryotes**	**Archaea**	**Bacteria**
Arginine	0.75%	0.75%	0.74%
Lysine	1.0%	1.05%	0.72%
Glutamic Acid	**1.28%**	**1.45%**	0.89%
Aspartic Acid	0.67%	0.65%	0.55%
Glutamine	0.57%	0.18%	0.42%
Asparagine	0.63%	0.37%	0.35%
Histidine	0.17%	0.09%	0.13%
Proline	0.83%	0.42%	0.43%
Tyrosine	0.23%	0.37%	0.21%
Tryptophan	0.04%	0.04%	0.04%
Serine	**1.76%**	0.90%	0.85%
Threonine	0.68%	0.60%	0.59%
Glycine	0.90%	1.10%	1.09%
Alanine	1.14%	**1.38%**	**1.92%**
Methionine	0.10%	0.11%	0.12%
Cysteine	0.10%	0.03%	0.04%
Phenylalanine	0.38%	0.38%	0.37%
Leucine	**1.87%**	**1.95%**	**2.02%**
Valine	0.80%	**1.31%**	1.03%
Isoleucine	0.61%	**1.23%**	0.74%

Legend: The dominant amino acids are leucine and serine (Eukaryotes), leucine and glutamic acid (Archaea), and leucine and alanine (Bacteria). Amino acids considered highly abundant are highlighted in bold.

### Prediction model

The prediction model shows a close correlation to the actual repeat distribution in many cases and in particular for short SAARs although there is a consistent slight under-estimation of the number of expected repeats. This would suggest that shorter repeats are mostly randomly distributed and that few of them are likely to be functionally significant. Short repeats are therefore likely to form part of the neutral drift of protein sequence evolution.

## Conclusion

COPASAAR provides an essential tool for the study of repeats in comparative proteomics. The ability to quickly analyse proteomes (and individual proteins) and to map the distribution and size of SAARs will hopefully benefit scientists from many different fields. COPASAAR will provide a useful resource for finding new protein families that can be used as species specific markers. Data on the evolution of repeats between species will also allow us to develop models of adaptive traits in proteomes. This will be particularly important in understanding the evolution of amyloid associated diseases.

## Availability and requirements

Online access to COPASAAR can be found at;



All of the source code for the project and the database files are also available from this site and are available under the GPL. Software requirements have been described above and non-academics should be aware of licensing restrictions regarding the use of the commercial software Perl ChartDirector.

## Authors' contributions

Both authors contributed to the design of COPASAAR and the underlying algorithms. The implementation of the system was carried out by DPD. Both DPD and ARD contributed to the final draft of the paper.

## Supplementary Material

Additional File 1 List of the proteomes in the database. Gives the species name and the FASTA identification number for the proteome Click here for file

## References

[B1] Pearson CE, Sinden RR (1998). Trinucleotide repeat DNA structures: dynamic mutations from dynamic DNA. Curr Opin Struct Biol.

[B2] Kruglyak S, Durrett R, Schug MD, Aquadro CF (2000). Distribution and abundance of microsatellites in the yeast genome can be explained by a balance between slippage events and point mutations. Mol Biol Evol.

[B3] LeProust EM, Pearso CE, Sinden RR, Gao XL (2000). Unexpected formation of parallel duplex in GAA and TTC trinucleotide repeats of Friedreich's ataxia. J Mol Biol.

[B4] Kashi Y, King D, Soller M (1997). Simple sequence repeats as a source of quantitative genetic variation. Trends Genet.

[B5] Alba MM, Santibanez-Koref MF, Hancock JM (1999). Conservation of polyglutamine tract size between mice and humans depends on codon interruption. Mol Biol Evol.

[B6] Alba MM, Guigo R (2004). Comparative analysis of amino acid repeats in rodents and humans. Genome Res.

[B7] Katti MV, Sami-Subbu R, Ranjekar PK, Gupta VS (2000). Amino acid repeat patterns in protein sequences: Their diversity and structural-functional implications. Protein Sci.

[B8] Fondon JW, Garner HR (2004). Molecular origins of rapid and continuous morphological evolution. Proc Natl Acad Sci U S A.

[B9] Marcotte EM, Pellegrini M, Yeates TO, Eisenberg D (1999). A census of protein repeats. J Mol Biol.

[B10] Djian P (1998). Evolution of simple repeats in DNA and their relation to human disease. Cell.

[B11] Sutherland GR, Richards RI (1995). The Molecular-Basis of Fragile Sites in Human-Chromosomes. Curr Opin Genet Dev.

[B12] Karlin S, Burge C (1996). Trinucleotide repeats and long homopeptides in genes and proteins associated with nervous system disease and development. Proc Natl Acad Sci U S A.

[B13] Bence NF, Sampat RM, Kopito RR (2001). Impairment of the ubiquitin-proteasome system by protein aggregation. Science.

[B14] Karlin S, Brocchieri L, Bergman A, Mrazek J, Gentles AJ (2002). Amino acid runs in eukaryotic proteomes and disease associations. Proc Natl Acad Sci U S A.

[B15] Hoffmann A, Sinn E, Yamamoto T, Wang J, Roy A, Horikoshi M, Roeder RG (1990). Highly Conserved Core Domain and Unique N-Terminus with Presumptive Regulatory Motifs in a Human Tata Factor (Tfiid). Nature.

[B16] Sun B, Fan W, Balciunas A, Cooper JK, Bitan G, Steavenson S, Denis PE, Young Y, Adler B, Daugherty L, Manoukian R, Elliott G, Shen WY, Talvenheimo J, Teplow DB, Haniu M, Haldankar R, Wypych J, Ross CA, Citron M, Richards WG (2002). Polyglutamine repeat length-dependent proteolysis of huntingtin. Neurobiol Dis.

[B17] Huntley MA, Golding GB (2002). Simple sequences are rare in the protein data bank. Proteins.

[B18] Nance MA (1997). Clinical aspects of CAG repeat diseases. Brain Pathol.

[B19] Pellegrini M, Marcotte EM, Yeates TO (1999). A fast algorithm for genome-wide analysis of proteins with repeated sequences. Proteins.

[B20] Advanced Software Engineering http://www.advsofteng.com/.

[B21] integr8 http://www.ebi.ac.uk/integr8.

[B22] Brendel V, Bucher P, Nourbakhsh IR, Blaisdell BE, Karlin S (1992). Methods and Algorithms for Statistical-Analysis of Protein Sequences. Proc Natl Acad Sci U S A.

[B23] Sim KL, Creamer TP (2002). Abundance and distributions of eukaryote protein simple sequences. Mol Cell Proteomics.

[B24] Sim KL, Creamer TP (2004). Protein simple sequence conservation. Proteins.

[B25] Ross CA, Margolis RL (2001). Huntington's disease. Clin Neurosci Res.

[B26] Cervantes-Kardasch VH, Garcia-Martinez E (2004). Molecular physiopathology of the spinocerebellar ataxia type 6 (SCA6). Rev Invest Clin.

[B27] Poirier MA, Jiang H, Ross CA (2005). A structure-based analysis of huntingtin mutant polyglutamine aggregation and toxicity: evidence for a compact beta-sheet structure. Hum Mol Genet.

[B28] Singh GP, Chandra BR, Bhattacharya A, Akhouri RR, Singh SK, Sharma A (2004). Hyper-expansion of asparagines correlates with an abundance of proteins with prion-like domains in Plasmodium falciparum. Mol Biochem Parasitol.

[B29] Kreil DP, Kreil G (2000). Asparagine repeats are rare in mammalian proteins. Trends Biochem Sci.

